# Triphenyl Phosphate Alters Methyltransferase Expression and Induces Genome-Wide Aberrant DNA Methylation in Zebrafish Larvae

**DOI:** 10.1021/acs.chemrestox.4c00223

**Published:** 2024-08-29

**Authors:** Chander K. Negi, Lucie Bláhová, Audrey Phan, Lola Bajard, Ludek Blaha

**Affiliations:** RECETOX, Faculty of Science, Masaryk University, Kotlarska 2, 611 37 Brno, Czech Republic

## Abstract

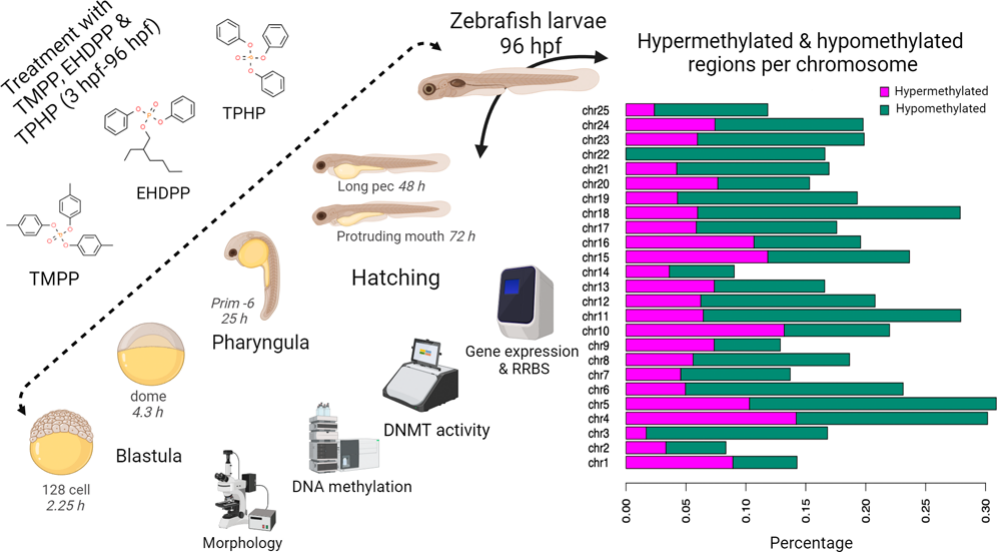

Emerging environmental contaminants, organophosphate flame retardants (OPFRs), pose significant threats to ecosystems and human health. Despite numerous studies reporting the toxic effects of OPFRs, research on their epigenetic alterations remains limited. In this study, we investigated the effects of exposure to 2-ethylhexyl diphenyl phosphate (EHDPP), tricresyl phosphate (TMPP), and triphenyl phosphate (TPHP) on DNA methylation patterns during zebrafish embryonic development. We assessed general toxicity and morphological changes, measured global DNA methylation and hydroxymethylation levels, and evaluated DNA methyltransferase (DNMT) enzyme activity, as well as mRNA expression of DNMTs and ten-eleven translocation (TET) methylcytosine dioxygenase genes. Additionally, we analyzed genome-wide methylation patterns in zebrafish larvae using reduced-representation bisulfite sequencing. Our morphological assessment revealed no general toxicity, but a statistically significant yet subtle decrease in body length following exposure to TMPP and EHDPP, along with a reduction in head height after TPHP exposure, was observed. Eye diameter and head width were unaffected by any of the OPFRs. There were no significant changes in global DNA methylation levels in any exposure group, and TMPP showed no clear effect on DNMT expression. However, EHDPP significantly decreased only DNMT1 expression, while TPHP exposure reduced the expression of several DNMT orthologues and TETs in zebrafish larvae, leading to genome-wide aberrant DNA methylation. Differential methylation occurred primarily in introns (43%) and intergenic regions (37%), with 9% and 10% occurring in exons and promoter regions, respectively. Pathway enrichment analysis of differentially methylated region-associated genes indicated that TPHP exposure enhanced several biological and molecular functions corresponding to metabolism and neurological development. KEGG enrichment analysis further revealed TPHP-mediated potential effects on several signaling pathways including TGFβ, cytokine, and insulin signaling. This study identifies specific changes in DNA methylation in zebrafish larvae after TPHP exposure and brings novel insights into the epigenetic mode of action of TPHP.

## Introduction

The widely used organophosphate flame retardants (OPFRs) have become pervasive in the environment. Several biomonitoring studies have detected their presence in the environmental matrix and human samples at relatively higher concentrations.^1−4^ Nevertheless, an extremely high concentration of OPFRs has also been reported in water bodies in several regions of the world.^5^ The ever-increasing presence of these chemicals may severely threaten the environment and human health. Moreover, humans are exposed to these environmental toxicants through several routes, including ingestion, inhalation of fine dust particles, food/dietary sources, etc.^6−8^ Human exposure to these chemicals has been associated with acute and long-term consequences.^9^ Several lines of evidence have shown a strong association between environmental exposure to chemicals, including flame retardants, and epigenetic alterations such as aberrant DNA methylation in humans.^10−14^

DNA methylation is one of the epigenetic modifications characterized by the covalent addition of the methyl (CH_3_) group at cytosine residues in the cytosine-phosphoguanine (CpG) dinucleotide sequence.^15^ DNA methylation is controlled by two enzymes: DNA methyltransferases (DNMTs) and ten-eleven translocation (TET) methylcytosine dioxygenase. DNMTs transfer a methyl group from *S*-adenosylmethionine (SAM) to the 5′ position of cytosine to generate 5-methylcytosine (5mC),^16^ whereas TET proteins are Fe(II)- and 2-oxoglutarate-dependent dioxygenases that catalyze the oxidation of 5mC forming intermediates such as 5-hydroxymethylcytosine (5hmC), 5-formyl cytosine (5fC), and 5-carboxyl cytosine (5caC), ultimately resulting in active demethylation of 5mC.^17^ DNA methylation can broadly be classified into two categories: DNA hypermethylation and DNA hypomethylation. Both can result in alteration in gene expression and are frequently observed in several pathological disorders.^18^ DNA hypomethylation causes gene reactivation and chromosomal instabilities, whereas DNA hypermethylation is involved in gene repression and chromosomal instabilities.^19,20^ DNA methylation is a heritable epigenetic modification that can occur in any DNA base.^21^ Although hypermethylation of promoter regions represses the gene expression, methylation in the gene body may increase the transcription by blocking the initiation of alternative promoters.^22^

The prenatal and early postnatal periods play a critical role in the developmental origin of health and diseases (DoHaD). During these times, environmental stressors, including exposure to chemicals in these crucial stages, can alter the developmental process, potentially leading to persistent effects that manifest as dysfunctional phenotypes later in life. Several studies have linked epigenetic alterations, such as DNA methylation, to the developmental origins of adult diseases.^23,24^ For instance, placental DNA methylation has been associated with numerous prenatal environmental exposures and with infant and childhood health outcomes, including fetal growth and birth weight neurodevelopment, etc.^25^ It has been reported that DNA methylation reprogramming occurs during embryonic development, and various environmental chemicals interfere with these processes, which may lead to adverse adult phenotypes.^26^ Several diverse classes of chemicals have already been known to affect DNA methylation, including essential vitamins and environmental pollutants, such as nickel and quinones, etc.^27−30^. Dysregulation in methylation patterns during early developmental phases could be related to late effects, such as obesity.^31^ Numerous studies have established a connection between epigenetic modifications, such as DNA methylation, and the developmental origins of adult diseases.^23,24^ These findings suggest that alterations in epigenetic mechanisms during critical periods of development may predispose individuals to various health issues later in their life. Therefore, it has become increasingly important to identify the epigenetic effects of environmental toxicants. Understanding how environmental pollutants influence DNA methylation and other epigenetic mechanisms during critical developmental windows can provide valuable insights into the potential long-term health impacts of these exposures. Zebrafish (*Danio rerio*) is a well-established test model in toxicology and developmental biology. Its utility in epigenetics is rapidly emerging due to the limitations of using rodent models because of economic and ethical issues.^32,33^ Moreover, zebrafish share 70% of their genes with humans and have proven to be a suitable model organism for both human and environmental toxicology in many studies, including epigenetics.^32^ Therefore, in the current study, we used the zebrafish model to identify the potential epigenetic effects and mechanisms of OPFRs: 2-ethylhexyl diphenyl phosphate (EHDPP), tricresyl phosphate (TMPP), and triphenyl phosphate (TPHP). We exposed the zebrafish embryo during the first 96 h and examined the effects on the morphology and general toxicity. We measured the transcriptional changes of DNMT1, DNMT3, DNMT4, DNMT5, DNMT6, DNMT7, DNMT8, TET1, TET2, and TET3 using the real-time quantitative polymerase chain reaction (RT-qPCR) and evaluated the overall effects on DNA methyltransferase (DNMT) enzyme activity. We measured the effects on global methylation and hydroxymethylation levels using the enzyme-linked immunosorbent assay (ELISA) and liquid chromatography–mass spectrometry (LC-MS). Finally, we measured the genome-wide methylation pattern using reduced-representation bisulfite sequencing (RRBS).

## Materials and Methods

### Zebrafish Maintenance and Exposure to Test Solution

**Figure 1:**
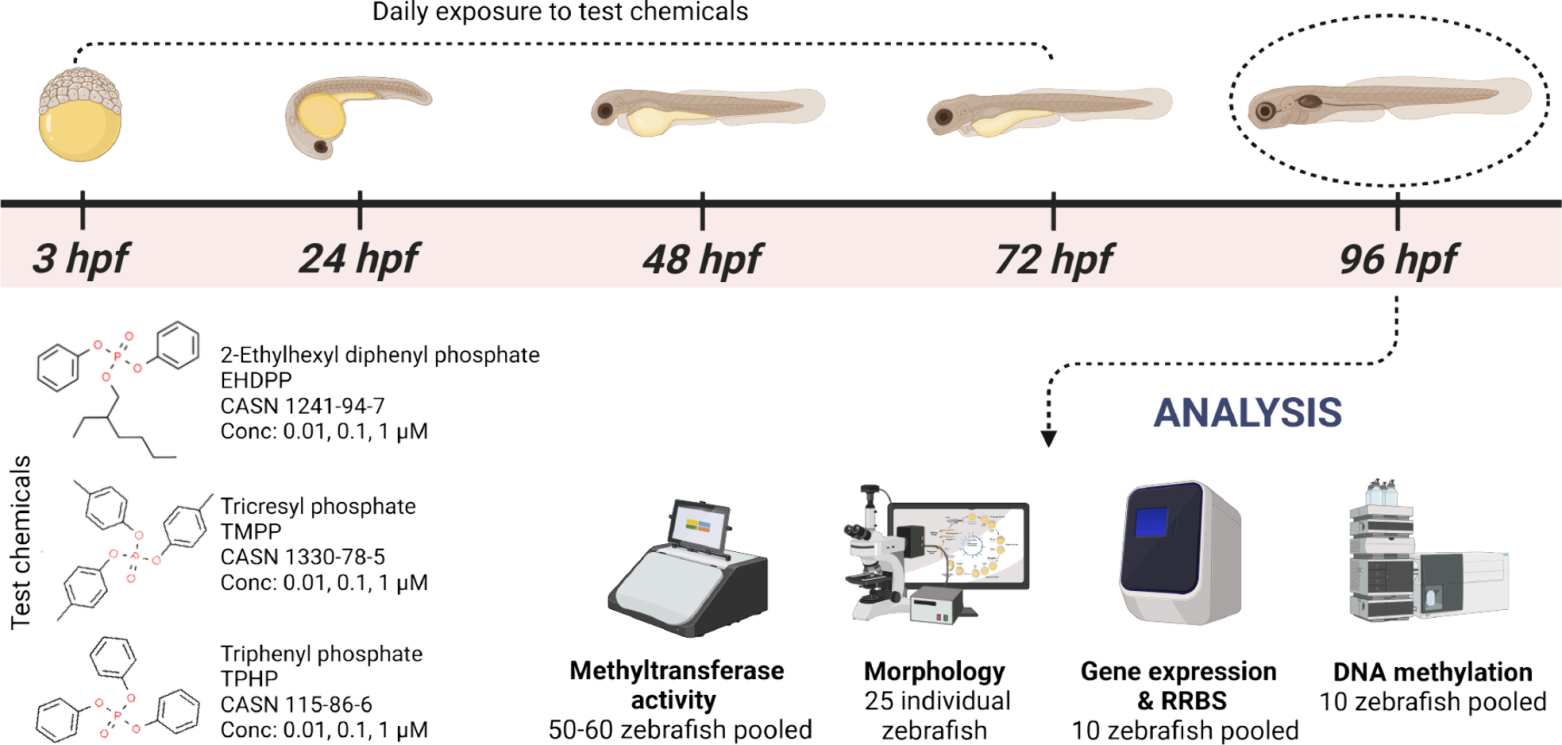
Experimental design. Wild-type zebrafish embryos were collected within 2 h post spawning. Fertilized and normally developing embryos at the blastula stage (3–4 hpf) were randomly distributed into glass beakers, each containing 100 embryos in 100 mL of ISO medium with varying concentrations of EHDPP, TMPP, and TPHP (0.01, 0.1, and 1 μM) or a solvent control (0.001% DMSO). Embryos were exposed daily until 96 hpf, with test solutions refreshed every 24 h. Survival and morphology were monitored at 24, 48, 72, and 96 hpf under white light microscopy. At 96 hpf, 25 larvae from the highest concentration of each treatment group were anesthetized, positioned for ventral and lateral imaging, and subjected to morphometric analysis. Ten zebrafish larvae were collected for DNA and RNA isolation, while 50–60 larvae were stored at −80 °C until nuclear protein extraction.

Wild-type adult zebrafish (*Danio rerio*) were maintained in an automatic flow-through aquarium system at 26 ± 0.5 °C under a 14:10 h light–dark photoperiod and fed two times a day with commercially available feeds and twice a week with live brine shrimp *Artemia salina*. Embryos were collected within 2 h post spawning, rinsed with distilled water, and maintained in standard fish medium^34^ at 26 ± 0.5 °C. Fertilized and normally developing embryos at 3–4 h postfertilization (hpf) at the blastula stage, as described by Kimmel et al.,^35^ were randomly distributed in glass beakers, each with 100 zebrafish embryos in 100 mL of ISO medium with different concentrations (0.01, 0.1, and 1 μM) of TMPP (TCI, Europe), EHDPP (TCI, Europe), TPHP (Sigma-Aldrich), and solvent control (SC, 0.001% DMSO) (Figure 1). The studied concentrations were based on a previous findings indicating nonlethal and nonteratogenic effects in zebrafish embryos during a 4 day exposure period. The lethal concentrations (LC50) values for TMPP, TPHP, and EHDPP were reported to be 9.52, 5.15, and 9.78 μM, respectively.^36^ Hence, the selected concentrations ensured that the observed effects were not confounded by mortality or severe developmental abnormalities, allowing us to focus on the molecular effects of OPFR exposure. The embryos were exposed daily until 96 hpf, with the test solution being replaced every 24 h. At the end of the exposure period (96 hpf), zebrafish larvae were collected and immediately processed for DNA and RNA isolation. For the extraction of nuclear protein, zebrafish larvae were stored at −80 °C until further analysis. Each experiment was independently repeated from different spawning events at least three times, unless otherwise specified. Chemical structures and Chemical Abstracts Service numbers (CASNs) of the studied compounds are presented in Figure 1. All of the chemicals were dissolved in dimethyl sulfoxide (DMSO) to prepare the stock solution (100 mM) and stored at −20 °C, and the amount of DMSO for exposure studies was not more than 0.001%.

### Morphology Observation

**Figure 2:**
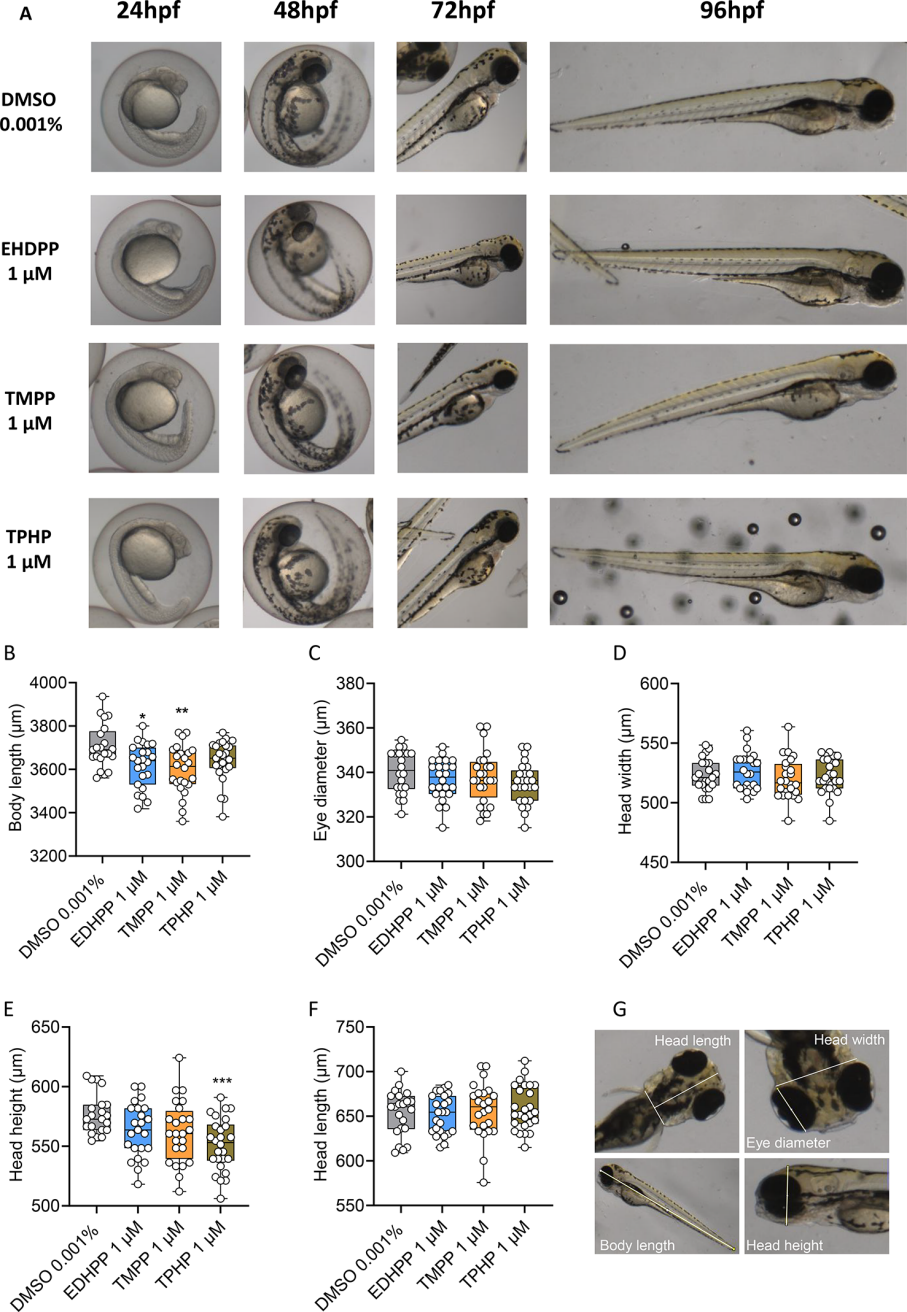
Effect of TMPP, TPHP, and EHDPP exposure on the morphology of zebrafish. (A) Representative bright field pictures of the embryos at 24, 48, 72, and 96 hpf. (B) Body length, (C) eye diameter, (D) head width, (E) head height, and (F) head length at 96 hpf. Data represent mean ± SEM of 25 zebrafish (*n* = 25) from one experiment. The asterisks indicate a significant difference from the control at *p* < 0.05 (*), *p* < 0.01 (**), *p* < 0.001 (***).

We measured the survival of embryos at different stages of development (24, 48, 72, and 96 hpf under white light microscopy; Nikon, Tokyo, Japan). In each stage, the total number of dead embryos were recorded. The indication of embryo death was defined based on opacity and egg clotting. At 96 hpf, 25 larvae from each treatment group were anesthetized with 100 μg/mL tricaine (MS-222, Sigma-Aldrich, St. Louis, MO, USA), and larvae were positioned on a glass Petri dish with 4% methylcellulose. Images of the larvae in ventral and lateral positions at 96 hpf were captured with a digital camera (AxioCam ICc 5, Zeiss, Jena, Germany) attached to a stereomicroscope (Stemi 508, Carl Zeiss, Oberkochen, Germany). Morphological measurements were conducted using ImageJ (https://imagej.net/ij/). Five morphometric parameters were evaluated for each zebrafish larva: body length, head width, head length, head height, and eye size. Examples of analyzed images for each end point are presented in Figure 2G.

### DNA Isolation and Measurement of Global Methylation Levels

Genomic DNA from 10 larvae per sample was isolated using a Quick DNA/RNA Miniprep Plus Kit (Zymo Research, USA) according to the manufacturer’s protocol. The quality of DNA was verified by using a nanodrop spectrophotometer (Thermo Fisher Scientific, USA). The global DNA methylation or the percentage of methylated cytosine in the genomic DNA (100 ng) was quantified using a MethylFlash Methylated DNA Kit (EpiGentek Group, Farmingdale, NY, USA), and the absorbance at 450 nm was measured in a BioTek plate reader (Winooski, VT, USA).

### Quantification of 5-mdC and 5-hmdC by LC-MS/MS

A total of 1 μg of DNA was digested to single nucleotides using DNA Degradase Plus (Zymo Research, USA) with 1× DNA degradase reaction buffer and 5U DNA degradase enzyme in a 25 μL volume with distilled water incubated at 37 °C for 4 h. The reaction was heat inactivated at 70 °C for 20 min, and the digested DNA was diluted 4 times with ultrapure water to make a final volume of 100 μL and transferred to GC vials stored at −20 °C for mass spectrometric analysis. DNA methylation was analyzed by an Acquity UPLC ultraperformance liquid chromatograph (Waters, Ireland) followed by a Xevo TQ-S tandem mass spectrometer (Waters, Ireland). The mobile phase consisted of 0.1% formic acid in water (A) and in acetonitrile (B), and the binary pump gradient was linear (3% to 80% B at 5 min). The flow rate was 0.2 mL/min, and 10 μL of each sample was injected. Analytes were detected in ESI positive ion mode, and the ionization parameters were as follows: capillary voltage, 2.5 kV; source temperature and desolvation temperature, 150 and 750 °C, respectively; cone gas flow, 150 L/h; cone voltages, 30 V; desolvation gas flow, 750 L/h; and collision gas flow, 0.15 mL/min. The methylation levels were determined from an external calibration curve (Software Mass Lynx, Manchester, UK). Concentrations of 2-deoxycytidine (dC), 5-methyl deoxycytidine (5-mdC), and 5-hydroxymethyl deoxycytidine (5-hmdC) were corrected for the internal standard 5-mdC d3 content. Quality assurance and quality control samples (5.0 ng/mL all analytes in 0.1% formic acid) were included in the analysis. The levels of 5-mdC and 5-hmdC in the DNA sample were expressed as a percentage of total cytosine content. The percentages of DNA methylation and hydroxymethylation were calculated according to the formulas DNA methylation % = 5-mdC/[5-mdC + 5-hmdC + dC] × 100% and DNA hydroxymethylation % = 5-hmdC/[5-mdC + 5-hmdC + dC] × 100%.

### Preparation of Nuclear Extract and Analysis of DNA Methyltransferase Enzyme Activity

The nuclear protein from zebrafish whole larvae was prepared using an EpiQuik Nuclear Extraction Kit I (Epigentek Group, Farmingdale, NY, USA). The nuclear extract was immediately quantified using standard Bradford’s Protein Assay Kit (Bio-Rad) and stored at −80 °C until further analysis. DNMT enzyme activity was measured in the nuclear extract using an EpiQuik DNMT Activity/Inhibition Assay Kit (EpiGentek Group, Farmingdale, NY, USA). The fluorescence was measured in a microplate reader (BioTek Synergy 5) at 450 nm.

### RNA Isolation and Quantitative Real-Time Polymerase Chain Reaction (RT-qPCR)

Total RNA from 10 larvae per sample was extracted using a Quick DNA/RNA Miniprep Plus Kit (Zymo Research, USA), and the RNA quality was verified using a nanodrop spectrophotometer (Thermo Fisher Scientific, USA). Total RNA (1 mg) was reverse transcribed to complementary DNA (cDNA) using a cDNA SensiFAST Kit (Bioline) in a final volume of 20 μL and stored at −20 °C until further analysis. The cDNA was amplified by RT-qPCR using GoTaq qPCR Master Mix (Promega) in a LightCycler 480 instrument (Roche). Primer sequences are listed in Table S1. The RT-qPCR reaction mixture comprised 1 μL of cDNA, 5 μL of GoTaq qPCR Master Mix 2×, and 0.4 μL of forward and reverse primers each (concentration 400 nM) in a final volume of 10 μL with PCR quality water (3.2 μL). The PCR conditions were as follows: 95 °C for 15 min, followed by 40 cycles of 10 s at 95 °C, 20 s at 60 °C, and 32 s at 72 °C. Melting curves were analyzed to validate the specificities of the PCR amplicons. The expression levels of DNMTs, including DNMT1, DNMT3, DNMT4, DNMT5, DNMT6, DNMT7, and DNMT8, and of TET1, TET2, and TET3 were examined. The expression levels of target genes were normalized to the geometric mean of two reference genes, βactin and eef1, and the relative mRNA levels of the gene of interest were quantified, according to Livak and Schmittgen’s method.^37^

### DNA Methylation Analysis by Reduced-Representation Bisulfite Sequencing (RRBS)

Genomic DNA was extracted from control and 1 μM TPHP exposed larvae and sent to CD Genomics (Shirley, NY, USA) for library preparation and sequencing. The genomic DNA was digested with MspI (NEB), followed by end preparation adaptor ligation using a Premium RRBS Kit (Diagenode). Size selection was performed using AMPure XP beads (Beckman Coulter, Inc.) to obtain DNA fractions of MspI-digested products enriching for the most CpG-rich regions in the 150–350 bp range. Subsequently, bisulfite treatment was conducted using a ZYMO EZ DNA Methylation-Gold Kit. The converted DNAs were then amplified by 12 cycles of PCR, using 25 μL of KAPA HiFi HotStart Uracil+ ReadyMix (2×) and 8 bp index primers with a final concentration of 1 μM each and cleanup using AMPure XP beads. The constructed RRBS libraries were quantified by a Qubit fluorometer with a Quant-iT dsDNA HS Assay Kit (Invitrogen) and sequenced on an Illumina Novaseq6000 platform using a paired-end 150 bp strategy in CD Genomics (Shirley, NY, USA). The FastQC tool (https://www.bioinformatics.babraham.ac.uk/projects/fastqc/) was used to perform statistical analysis on the quality of the raw reads. Then, sequencing adapters and low-quality sequencing data were removed by Trimmomatic (version 0.36).^38^ The BSMAP software was used to map the bisulfite sequence to the reference genome with parameters *n* 0 −*g* 0 −*v* 0.08 −*m* 50 −*x* 1000. The statistical information on the alignment was collected. Only the unique mapped reads were kept for the following analysis, and methylated cytosines with a sequence depth coverage of at least 5 were used. If the base on the alignment is C, then methylation occurs; conversely, if the base on the alignment is T, then no methylation occurs. The methylation levels of individual cytosines were calculated as the ratio of the sequenced depth of the ascertained methylated CpG cytosines to the total sequenced depth of individual CpG cytosines, i.e., ML = mC/(mC + umC), where ML is the methylation level, and mC and umC represent the number of reads supporting methylation C and the number of reads supporting unmethylated C, respectively. The software metilene (version 0.2-7) was used to identify the differentially methylated region (DMR) by a binary segmentation algorithm combined with a two-dimensional statistical test (parameters: −*M* 300 −*m* 5 −*d* 0.1 −*t* 1 −*f* 1 −*v* 0.7). Gene Ontology (GO, http://www.geneontology.org/) enrichment analysis of DMR-related genes (genes found within 500 bp upstream and downstream of DMRs are identified as DMR genes) was applied to uncover biological processes of interest; we chose to deem pathways with a *q* value of ≤0.05 as significantly enriched with DMR-related genes. Based on the results of the DMR annotation and the database of Kyoto Encyclopedia of Genes and Genomes (KEGG), functional enrichment analysis was performed on genes whose gene body and upstream and downstream regions (upstream 2k, gene body, and downstream 2k) overlap with DMR.

### Statistical Analysis

All data are expressed as the mean ± the standard error of the mean (SEM) of three independently repeated experiments. Statistical analysis was performed using GraphPad Prism version 9 for Windows (GraphPad Software, La Jolla, CA, USA, www.graphpad.com). One-way analysis of variance (ANOVA) test was performed for comparison between the groups, and a *p* < 0.05 value was considered statistically significant.

## Results

### Effects of OPFR Exposure on Zebrafish Development

The morphology of zebrafish was assessed by measuring five key parameters: body length, eye diameter, head width, head height, and head length. The results, illustrated in the box plots in Figure 2, reveal distinct morphological changes across different treatment groups. Notably, the body length of zebrafish showed significant changes across the different treatment groups. The TMPP and EHDPP (1 μM) treatments slightly but significantly reduced the zebrafish body length, whereas the zebrafish body length of the TPHP treatment did not significantly differ from that of the control. There were no significant differences in the eye diameter between the treatment groups. The median eye diameter remained consistent across all groups, indicating that these treatments did not impact the eye size. Additionally, the head width and head length of zebrafish were unaffected by exposure to TMPP, EHDPP, and TPHP. However, the head height was significantly decreased after TPHP exposure, while TMPP and EHDPP treatments did not show any significant differences in head height. TMPP and EHDPP treatments primarily affect body length; TPHP specifically impacts head height without altering other morphological parameters, suggesting different chemical exposures can result in distinct morphological changes in zebrafish.

### Effects on DNMT and TET Gene Expression

**Figure 3:**
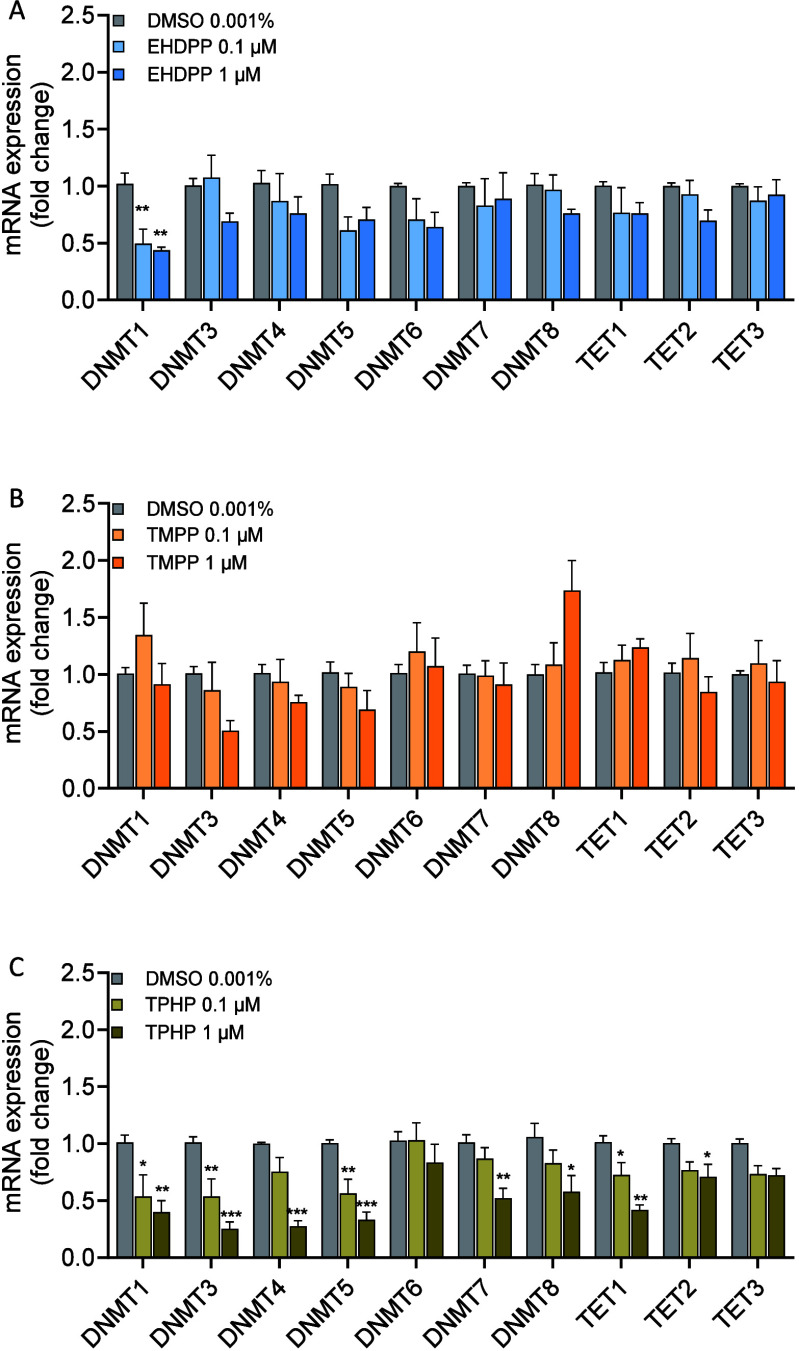
Effect on transcription of genes related to methylation. DNMT1, DNMT3, DNMT4, DNMT5, DNMT6, DNMT7, DNMT8, and TET1, TET2, and TET3 of zebrafish larvae exposed to (A) TMPP, (B) EHDPP, and (C) TPHP for 96 hpf. Data represent mean ± SEM of three independent experiments (*n* = 3). The asterisks indicate a significant difference from the control at *p* < 0.05 (*), *p* < 0.01 (**), *p* < 0.001 (***).

To further understand the molecular impact of OPFR exposure, we examined the gene expression levels of DNMTs and TET enzymes, which play pivotal roles in the regulation of DNA methylation and hydroxymethylation. Our results indicate that TPHP exposure significantly decreased the expression of several DNMTs, including de novo DNMT3B (orthologues DNMT3, DNMT4, DNMT5, and DNMT7) and DNMT3A (orthologues DNMT8), as well as maintenance DNMT1. Additionally, TPHP reduced the levels of TET1 and TET2 genes (Figure 3). In contrast, EHDPP exposure significantly decreased the expression of only DNMT1. No significant changes in the expression of any DNMT and TET genes were noted in the TMPP treatment group. This observation implied that TPHP might potentially affect DNA methylation during zebrafish developmental phases. To investigate this, we examined the impact of these OPFRs on DNMT activity in nuclear extracts from whole zebrafish larvae. However, no significant changes in DNMT enzyme activity were observed across the treatment groups (Figure S1).

### Effects on Global DNA Methylation and Hydroxymethylation

**Figure 4:**
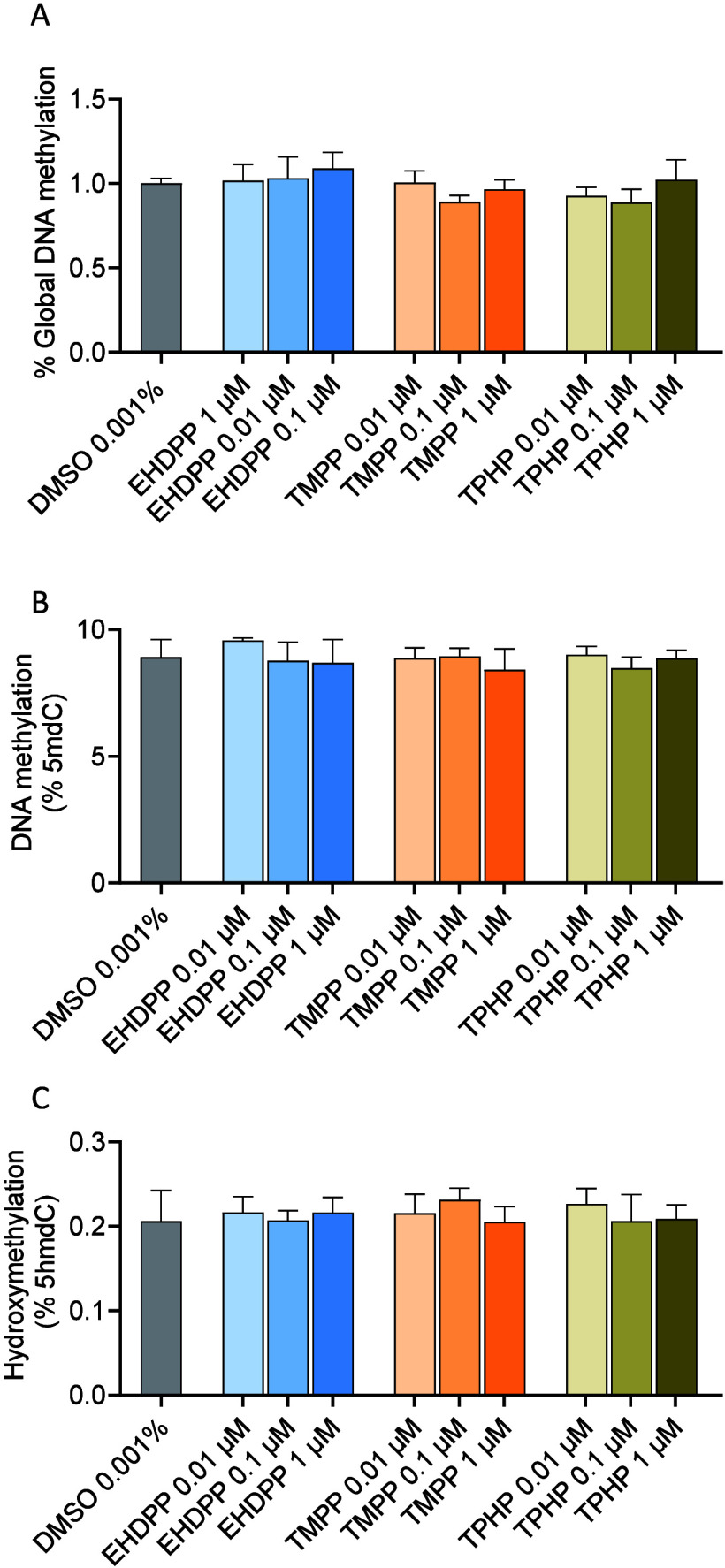
Effect on global DNA methylation and hydroxymethylation of zebrafish larvae exposed to TMPP, EHDPP, and TPHP for 96 hpf: (A) ELISA method and (B, C) LC/MS-based methods. Data represent mean ± SEM of three independent experiments (*n* = 3).

Exposure to OPFRs did not induce significant changes in the global DNA methylation level in exposed zebrafish larvae, as identified using ELISA (Figure 4A) and LC/MS-based methods (Figure 4B). Neither did it induce any significant changes in the global hydroxymethylation levels (Figure 4C). As the global methylation level indicates the overall methylation of CpG sites in the entire genome, this approach may lack sufficient sensitivity or specificity to capture changes to certain chromosome regions or individual genes. Thus, localized epigenetic modifications might not be captured by these global analyses, necessitating a more targeted approach to fully understanding the effects on specific genomic sites.

### Effects on Whole Genome-Wide DNA Methylation Pattern

**Figure 5:**
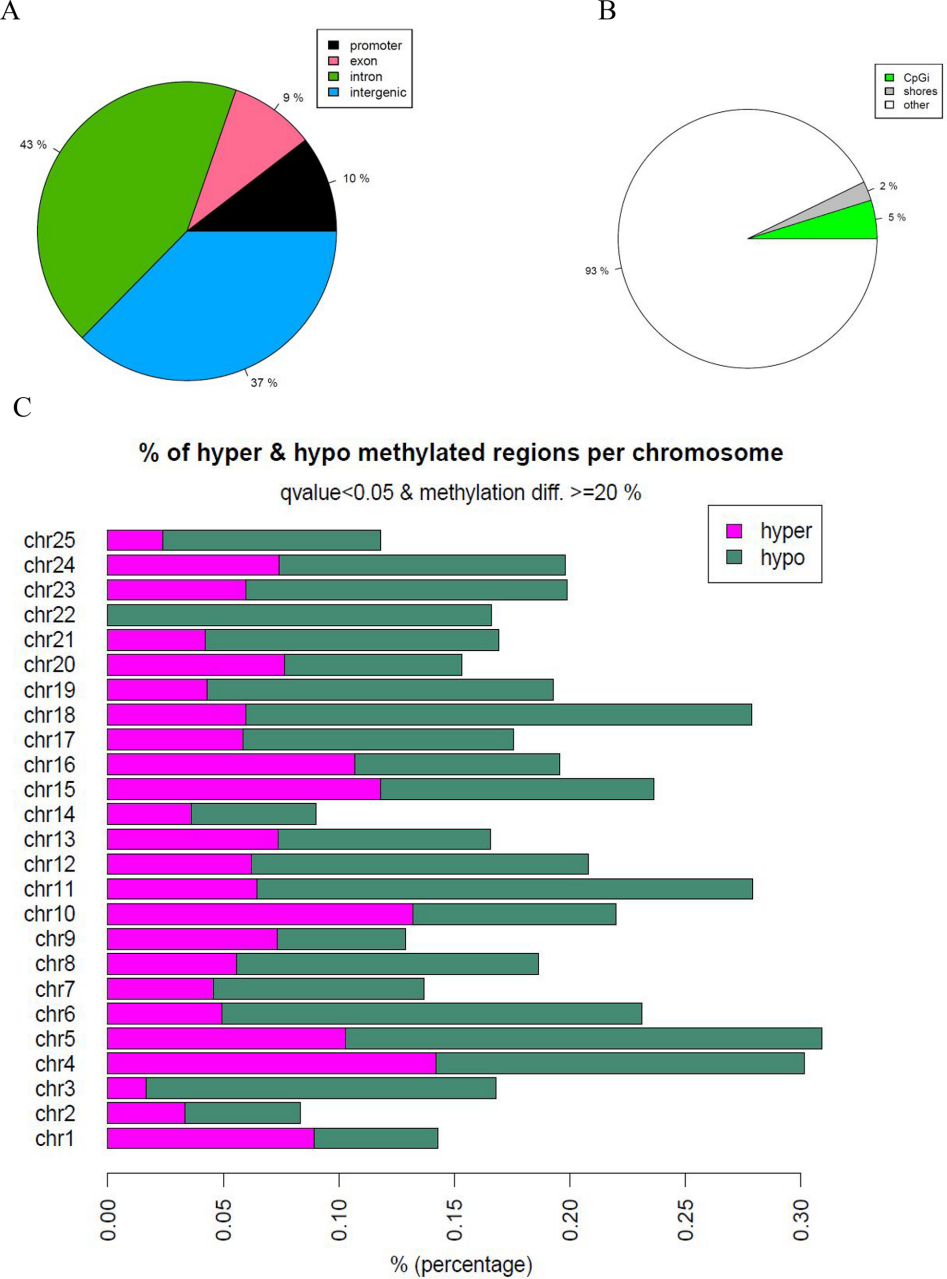
Genome-wide profile of CpG methylation. (A) The proportion of DMR located in exons, introns, promoters, and intergenic regions. (B) The proportion of DMR located in CpGi, CpG shores, and other CpG-containing sequences. (C) Horizontal bar plot shows the percentage of hyper- and hypomethylation per chromosome.

Since we observed a significant decrease in the DNMTs and TET expression after TPHP exposure, we used RRBS to identify the genome-wide alteration in methylation profiles of zebrafish larvae exposed to 1 μM TPHP. Global methylation analysis offers a broad perspective on genome-wide methylation levels but may overlook regional variations and gene-specific changes. In contrast, chromosome-specific methylation analysis provides a more precise and targeted approach, enhancing the detection of dynamic methylation patterns within specific genomic regions and individual genes. With exposure to TPHP-induced chromosome-specific alteration in the cytosine methylation within the CpG context at 96 hpf (Figure 5), we identified a total of 249 DMRs with methylation differences >20% at a false discovery rate (FRD) corrected *p* < 0.05 (Supporting Information, Table S2). These DMRs included 87 hypermethylated and 162 hypomethylated DMRs. Differential methylation of CpG occurred mainly in the introns (43%) and intergenic regions (37%), while 9% and 10% are in the exons and promoter regions, respectively. The pie chart in Figure 5A indicates the methylated sites in each genomic element, e.g., transcription regulatory regions such as the promoters, gene body (exons and introns), and intergenic region. Table S2 lists the significant hypomethylated and hypermethylated DMRs for TPHP-treated zebrafish larvae vs control.

### Pathway Analysis of DMR-Associated Genes

**Figure 6:**
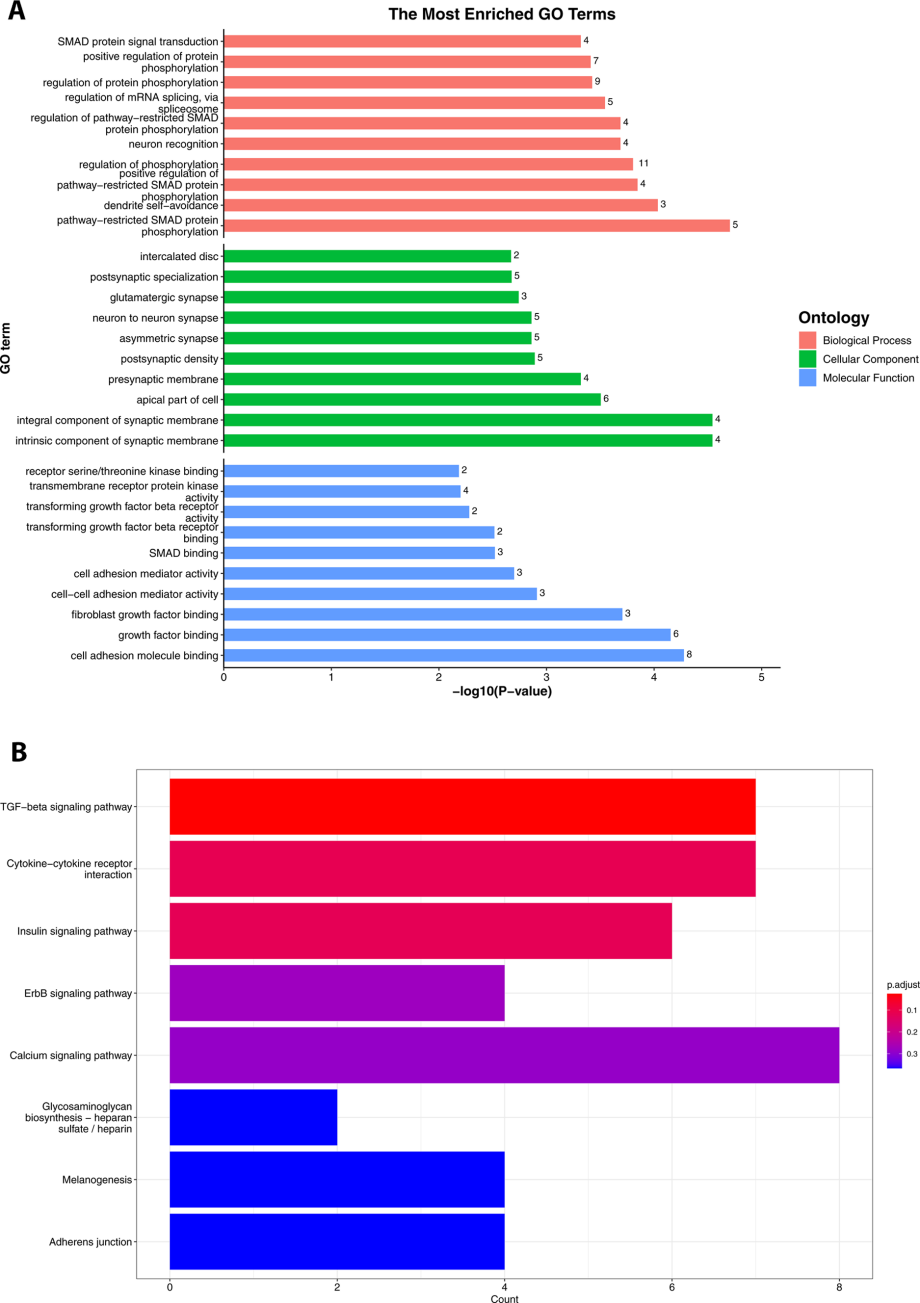
(A) GO analysis of the topmost enriched pathways from biological processes, cellular components, and molecular functions. (B) KEGG enrichment analysis of DMR-associated genes.

To identify if the DMRs are associated with specific biological processes (BP), cellular components (CC), and molecular functions (MF), we used Gene Ontology (GO) enrichment analysis, which allowed us to biologically contextualize genes found to be differentially methylated in the intron, exon, and promoter regions of the whole zebrafish larvae. We analyzed all hypermethylated and hypomethylated DMRs and observed that exposure to TPHP enriched the top 10 pathways involved in transcriptional and developmental processes, including SMAD signaling, receptor signaling, and neuronal developmental and metabolic processes (Figure 6A). KEGG enrichment analysis indicated TPHP-mediated statistically significant enhancements in transforming growth factor beta (TGFβ) signaling, cytokine, insulin signaling, and ErbB-1 (epidermal growth factor receptor) signaling (Figure 6B). Table S3 presents the topmost significantly enriched pathways after the enrichment analysis of all DMR-associated genes.

## Discussion

In this study, we analyzed DNA methylation levels in zebrafish larvae exposed to the OPFRs EHDPP, TMPP, and TPHP, with the primary objective of exploring potential epigenetic effects by assessing changes in global DNA methylation patterns. To investigate these changes, we employed various techniques, including ELISA and LC/MS-based methods. However, neither method detected significant alterations in global DNA methylation or hydroxymethylation levels. This outcome aligns with previous observations suggesting aggregated global cytosine methylation analyses may lack the sensitivity to detect spatially resolved, position-specific effects.^39^ Additionally, global methylation analysis provides a broad assessment of methylation across the entire genome; it may fail to capture variations and alterations specific to certain chromosomal regions or individual genes. This limitation could explain our inability to detect changes in the aggregate or global methylation levels. To further explore the potential epigenetic effects of these exposures, we conducted transcriptional analyses of DNMTs and TETs. This analysis revealed that TMPP exposure had no significant impact on the expression of DNMTs and TETs, while EHDPP exposure specifically led to a decrease in the level of DNMT1 expression. In contrast, TPHP exposure resulted in a marked reduction in both DNMT and TET expressions. The decrease in the gene expression of DNMTs and TETs were also observed in human liver (HepG2) cell culture (see Figure S2), further emphasizing the potential role of TPHP in epigenetic regulation. Additionally, a recent study also reported a significant decrease in fetal DNA methylation following in utero exposure to 50 mg/kg TPHP to pregnant C57Bl/6 mice.^40^ These findings collectively suggest that TPHP may influence DNA methylation processes. Therefore, to further explore this, we employed RRBS, a more sensitive, robust, and powerful method for detecting DNA methylation variations on a genome-wide scale. This approach allows for a comprehensive analysis of methylation patterns, providing deeper insights into the epigenetic impact of TPHP exposure. As expected, exposure to 1 μM TPHP induced a chromosome-specific alteration in cytosine methylation within the CpG context in zebrafish larvae. The genome-wide analysis identified that after exposure to TPHP, 162 genes were hypomethylated, while 87 genes were hypermethylated. Differential methylation occurred mainly in the gene body, promoter, and intergenic regions. It is recognized that DNA methylation at the promoter is widely associated with transcriptional repression, while hypomethylation induces transcriptional expression. However, studies indicate that methylation outside promoter regions, such as gene body methylation, also plays a prominent role in gene regulation.^41−45^

We identified differential methylation predominantly in the gene body and only 10% differential methylation in the promoter region. Among the differentially methylated genes are ACACA, PLIN2, CRLS1, SOX6, GSK3β, and TGFβ2, which have widely been known to affect several metabolic signaling pathways. These genes are integral to lipid metabolism, energy homeostasis, and cellular function, suggesting that TPHP exposure may have an effect on metabolic health. ACACA, acetyl-CoA carboxylase alpha, is a fatty acid biosynthesis gene and is also shown to be upregulated in low-fat diet-fed mice after TPHP exposure.^46^ PLIN2, or perilipin 2, is an adipogenesis gene crucial for lipid storage that might impair lipid droplet formation and mobilization, contributing to conditions such as insulin resistance and metabolic syndrome.^47^ CRLS1 is a cardiolipin synthesis gene and a phospholipid of the mitochondrial membrane and plays an important role in mitochondrial function and numerous cellular processes, including cardiovascular health. Correspondingly to our observations, another recent study reports that TPHP exposure enhanced cardiolipin levels, leading to thrombosis in zebrafish larvae.^48^ Aberrant methylation of CRLS1 could also be suggested as one of the potential causes for elevated levels of cardiolipins in zebrafish larvae. Other important differentially methylated genes after TPHP exposure included SOX6 [SRY (sex determining region Y)-box 6], which is a transcription factor that has recently been reported to contribute to the developmental origins of obesity, as shown using human, mouse cell culture, and zebrafish larvae potentially by regulating PPAR γ, C/EBP, and WNT signaling pathways.^49^ Further, glycogen synthase kinase 3 beta (GSK3β) is a serine/threonine kinase that plays a key role in various cellular processes, including development, cell proliferation, and signal transduction. GSK3β has been implicated in the regulation of glucose metabolism and insulin signaling.^50^ Abnormal GSK3β expression can disrupt insulin signaling, potentially contributing to insulin resistance and metabolic disorders. TGFβ2 plays a diverse role across different cell types, mainly signals through a downstream mediator, transcription factors called SMAD proteins.^51^ The TGFβ family of growth factors has been shown to play an important role in pancreatic β-cell identity and function or homeostasis.^52,53^ Moreover, transgenic overexpression of TGFβ decreased the development of exocrine pancreas and islets, induced β-cell hypoplasia, reduced insulin secretion, and impaired glucose tolerance in mouse models.^54,55^

The collective methylation changes in these genes indicate that TPHP exposure can potentially disrupt multiple aspects of lipid metabolism and energy homeostasis, which could contribute to the development and progression of metabolic diseases. Interestingly, TPHP has also been noted to affect lipid metabolism in several studies involving human liver cells and zebrafish. For instance, studies have demonstrated that TPHP exposure leads to altered lipid accumulation and changes in the expression of genes involved in lipid metabolism in human liver cells.^56−58^ Similarly, zebrafish studies have shown that TPHP disrupts normal lipid metabolic processes in zebrafish liver.^59^ These findings support the idea that TPHP-induced epigenetic modifications in key metabolic genes may be a crucial mechanism underlying the observed metabolic disruptions. The consistency of these effects across different models, including human cells and zebrafish, underscores the potential health risks associated with TPHP exposure, particularly in relation to metabolic health.

We used pathway analysis from the differentially methylated genes to get broad insights into affected signaling pathways. The GO analysis revealed a TPHP-mediated potential disruption of several biological cellular and molecular functions. For instance, they mainly affected the receptor signaling pathways and metabolic and neurological processes. These findings correspond well with previous studies that identified TPHP as a potential neurotoxicant in several experimental animals, including Chinese rare minnows, marine medaka, zebrafish larvae, etc.^60−63^ Our analysis indicates that TPHP exposure disrupts pathways and signaling involved in neuronal development and regulation, providing additional evidence of the potential neurotoxic effects of TPHP that might be linked to epigenetic mechanisms. The observed decrease in head height in our study suggests that TPHP may interfere with cranial development, potentially affecting critical aspects of neurological function and development, which is consistent with previous studies reporting TPHP-induced developmental neurotoxicity.^60^

Furthermore, KEGG pathway analysis using a *p* value threshold of less than 0.05 identified several enriched pathways. These include the TGFβ signaling pathway, cytokine–cytokine receptor interaction, insulin signaling pathway, ErbB signaling pathway, calcium signaling pathway, glycosaminoglycan biosynthesis - heparan sulfate/heparin, melanogenesis, and adherens junction. The enrichment of these pathways highlights the potential for TPHP to interfere with a wide range of biological processes, from cellular communication and inflammatory responses to glucose/lipid metabolism and cell growth. These findings align with previous studies that show TPHP-mediated defects in the calcium-dependent signaling pathways, leading to abnormal learning and memory behaviors by perturbing synaptogenesis and neurotransmission in wild-type (C57BL/6) mice.^64^ Interestingly, in alignment with our observations, TPHP-mediated enhancement in the adherens junction pathway has also been reported for zebrafish^65^ and human Hep3B cells.^66^ Moreover, TPHP also affected the carbohydrate metabolism pathway, mainly glycosaminoglycans, as reported in previous studies in zebrafish.^59,65^ TPHP-mediated insulin resistance in rodents has also been reported in a few publications.^46,67^ These observations further strengthen the potential role of epigenetic regulatory mechanisms in TPHP-induced adverse biological responses in zebrafish. The implications of these disruptions are significant, as they suggest that TPHP may contribute to the development of metabolic diseases, such as diabetes and obesity.

DNA methylation is an epigenetic modification regulated by DNMTs and TETs. In zebrafish, there are seven DNMT genes: one DNMT1, which is similar to human maintenance DNMT1, and six DNMT paralogues similar to human de novo methyltransferases DNMT3A (zebrafish orthologues DNMT6 and DNMT8) and DNMT3B (zebrafish orthologues DNMT3, DNMT4, DNMT5, and DNMT7).^68^ DNMT3A and DNMT3B are responsible for the methylation of unmethylated DNA, and DNMT1 is a methylation maintenance enzyme. When the activity of DNMTs is blocked or decreased, passive DNA demethylation occurs where 5mC is passively converted back to cytosine. Meanwhile, TETs catalyze the oxidation of 5mC, ultimately resulting in the active demethylation of 5mC.^69^ It has been noted in this study that TPHP significantly reduced the methyltransferase expression for DNMT3B (orthologues DNMT3, DNMT4, DNMT5, DNMT7) and DNMT3A (orthologues DNMT8); it nevertheless also decreased DNMT1, which indicates a passive demethylation process after TPHP exposure. However, in the present study, TMPP did not induce any significant changes in DNMT and TET expression, but EHDPP significantly decreased the expression of DNMT1. These DNMTs are localized in a tissue-specific pattern in zebrafish and play an important role in the developmental process. For instance, DNMT3AA, DNMT3AB, and DNMT4 play important roles in the formation of various organs, including the brain, kidney, digestive organs, and hematopoietic cells, as well as in the differentiation of blastema cells.^70^ More recently, it has been demonstrated that DNMT1 is required for the development of zebrafish auditory organs.^71^ Our morphological analysis revealed a slight but significant reduction in body length following exposure to TMPP and EHDPP, while TPHP primarily resulted in a notable decrease in head height. These findings align with previous research that observed reductions in body length in zebrafish exposed to EHDPP.^72^ Similarly, zebrafish embryos exposed to different isomers of TMPP exhibited significant reductions in survival rates, body length, and swimming abilities.^73^ The observed decrease in body length after TMPP and EHDPP exposure and the reduction in head height after TPHP exposure suggest potential disruptions in growth and development, which might arise from several toxicological mechanisms. These could include direct interference with cellular processes, endocrine disruption, or oxidative stress. Notably, TPHP exposure led to a marked reduction in DNMT and TET expression, which are crucial for maintaining DNA methylation patterns, regulating gene expression, and maintaining genomic stability. The decrease in DNMT expression observed after TPHP exposure could impair these regulatory functions, potentially leading to aberrant gene expression and developmental impairments. TPHP-mediated reduction in DNMT expression may thus be linked to disruptions in growth and development, which have been observed previously in several studies. For example, disturbance in ocular development and muscular organization,^74^ visual function and impaired retinal development,^75^ cardiac malformation,^76^ and most importantly, developmental neurotoxicity.^36^ It is not known whether these alterations in the methylation patterns are caused by compounds directly or as a consequence of their upstream toxicological mode of action. Moreover, DNMT3 plays a crucial role in neurogenesis in zebrafish, as evidenced by studies showing that DNMT3 knockdown leads to significant neurogenesis defects and a smaller head size in zebrafish embryos.^77^ These findings suggest a direct link between DNMT3 activity and the development of the head morphology in zebrafish. Specifically, the reduction in head height was observed after TPHP exposure, along with decreased DNMT expression, which indicates a potential epigenetic mechanism underlying TPHP-induced neurodevelopmental toxicity.

In summary, our study did not observe a significant change in global DNA methylation levels of zebrafish larvae after an initial 96 h exposure to TMPP, EHDPP, and TPHP. In contrast, chromosome-specific methylation analysis after TPHP exposure shows significant variation, which can be explained by several factors related to the complexity of DNA methylation patterns and the limitations of global methylation analysis. While global methylation analysis provides an overall measure of methylation across the entire genome, chromosome-specific methylation analysis provides high-resolution base-pair-level data, allowing for the precise identification of methylated cytosines in specific genomic regions across the entire genome. It detects regional variations in methylation that may be averaged out in global analyses and provides gene-specific insights into regulatory regions. However, due to the high cost and resource-intensive nature of RRBS, we were unable to extend our methylation analysis to include EHDPP and TMPP. The decision to prioritize TPHP was based on its widespread use as a replacement for more toxic flame retardants such as PentaBDE and its associated ecological and health risks. TPHP has been extensively studied due to its potential to induce developmental abnormalities, cardiotoxicity, and oxidative stress in aquatic organisms, particularly zebrafish, at environmentally relevant concentrations. Furthermore, TPHP exposure has been linked to disruptions in gene expression pathways including those involved in endocrine function, metabolism, and neurodevelopment. The observed epigenetic effects following TPHP exposure align with previous studies conducted in mice and fish cell lines.^40,78^ These findings suggest that TPHP induces similar epigenetic changes across different species, highlighting the potential widespread impact of this chemical on genetic regulation. These alterations could have significant implications for aquatic ecosystems and human health, suggesting that TPHP-mediated epigenetic effects warrant further investigation. Since DNA methylation patterns can be tissue- or cell-type-specific, the observed significant variations in chromosome-specific methylation after TPHP exposure may be specific to the tissue or cell type, which could not be captured in the present study with the whole zebrafish larvae, representing the average or most prominent effects across the multiple cell types.

## Conclusions

We identified the alteration in chromosome-specific DNA methylation patterns during the embryonic development of zebrafish exposed to the widely used environmental toxicant TPHP. Notably, TPHP-induced differential methylation was predominantly observed in the gene body. Enrichment analysis of DMR highlighted key pathways and processes associated with metabolism and neurodevelopment that may be affected by TPHP. Additionally, the transcriptional analysis revealed a decrease in the DNMT and TET expression alongside a notable decrease in head height, suggesting a mechanistic link between DNMTs and neurodevelopment. Future research should focus on the transgenerational impact of TPHP-mediated epigenetic modification-associated adverse effects and tissue-specific effects on the methylome. It would be interesting to know whether methylation changes caused by TPHP exposure during early development persist to later stages. Such studies will enhance our understanding of the long-term and potentially heritable consequences of environmental toxicant exposure on epigenetic regulation and organismal health.

## Supporting Information Available

All DMRs with methylation differences >20% at a false discovery rate (FRD) corrected *p* < 0.05 and complete lists of significantly enriched KEGG and GO pathways (XLSX)
Additional information regarding RT-qPCR primer sequences, human liver (HepG2) cell culture study, and analysis methods (PDF)
