# Supplementary material for: Triphenyl Phosphate Alters Methyltransferase Expression and Induces Genome-Wide Aberrant DNA Methylation in Zebrafish Larvae
Source: Chem Res Toxicol. 2024 Aug 29;37(9):1549–61. doi: 10.1021/acs.chemrestox.4c00223 (PMC11409374; doi:10.1021/acs.chemrestox.4c00223)
Supplement: Supplementary file 2 — tx4c00223_si_002.pdf [file tx4c00223_si_002.pdf]

Supporting information

Triphenyl phosphate alters methyltransferase expression and induces genome-wide aberrant DNA methylation in zebrafish larvae

Chander K. Negi*, Lucie Bláhová, Audrey Phan, Lola Bajard, Ludek Blaha

RECETOX, Faculty of Science, Masaryk University, Kotlarska 2, 611 37 Brno, Czech Republic

Address for correspondence:

Chander K. Negi, Ph.D.

Email: chander.negi@recetox.muni.cz, chandernegi09@gmail.com

RECETOX, Faculty of Science, Masaryk University,

Kotlarska 2, 611 37 Brno, Czech Republic.

Number of pages: 5

Number of tables: 1

Number of figures: 2

Table of Contents

Cell culture chemical exposure and RT-qPCR 2
Table S1. List of oligonucleotides 3
Figure S1. Effect of OPFRs on DNA methyltransferase activity of zebrafish larvae...4
Figure S2. Effects of TPHP exposure on viability, DNMT and TET gene expression in HepG2 cells.. 5

1 Cell culture chemical exposure and RT-qPCR

Human hepatocellular carcinoma HepG2 cells, obtained from the American Type Culture Collection (ATCC, Manassas, VA, USA), were cultured in Minimum Essential Media (MEM) (Gibco, NY, USA) supplemented with sodium pyruvate, non-essential amino acids, 1.50 g/L NaHCO3, and 10% fetal bovine serum. Cells were maintained at 37°C in a humidified atmosphere of 95% air and 5% CO2. Cell viability analysis was done using a combination of three classical dye methods; neutral red, CFDA-AM, and Resazurin after 24 h exposure with TPHP concentrations ranging from 0.1 to 100 μM. For RNA isolation, 100,000 cells per well were seeded into a 24-well plate. After 24 hours, the cells were treated with non-cytotoxic concentrations of TPHP (1 and 10 μM) for an additional 24 hours. Post-exposure, total RNA was extracted using the RNeasy Mini Kit (Qiagen Inc., Mississauga, ON) following the manufacturer's instructions. RNA quality and quantity were assessed spectrophotometrically at 260 nm using a Nanodrop spectrophotometer (Thermo Fisher Scientific, USA), with an A260/280 ratio between 1.8 and 2.0 considered acceptable for RNA purity. RNA was then reverse-transcribed using the cDNA SensiFAST™ cDNA synthesis kit (Bioline). The resultant cDNA was amplified via RT-qPCR with SYBR™ Green PCR master mix (Thermo Fisher Scientific, Waltham, MA) on a Roche 480 LightCycler (Roche, Basel, Switzerland). Primer sequences are listed in Table S2. RT-qPCR conditions were: initial denaturation at 95°C for 2 minutes, followed by 40 cycles of 95°C for 5 seconds, 60°C for 10 seconds, and 72°C for 20 seconds, with a melt curve analysis of 95°C for 5 seconds and 55°C for 1 minute. Specificity of the RT-qPCR products was confirmed by melt curve analysis. Gene expression levels were normalized to two reference genes: eukaryotic elongation factor 1 gamma-like protein (EEF-1) and malate dehydrogenase 1 (MDH1) mRNA levels. Relative mRNA levels of target genes were quantified using the method described by Livak and Schmittgen's (2001) method.

Table S1. List of oligonucleotides used in this study**.**

Genes	Forward sequence	Reverse sequence
Zebrafish		
DNMT1	GAGGAGGATGTGTTGCCAGTTA	CCTCATTTTCCACACGCACTTT
DNMT3	TAGAGTCATGTTGAACTGGGCC	TCAGGTCCAGAGATTCAGGGAT
DNMT4	AAGATTTACCCTGCAGTCCCAG	CTCGCATACTTCTGACGCAATG
DNMT5	TTATCCACCCACTGTTCGAAGG	ATGACCACACAGAATGACCTCC
DNMT6	GTGTGGGGAAAGTTACGAGGAT	TGCTTATTGTAGGTTGGCTGGT
DNMT7	AGGCAGCTTTTCGGGATTTAGA	CGATTTCTTGACCATCACGAGC
DNMT8	CTTTGCCTGTTAATGAAGCCCC	TGTGAAGTGTCCTGTGGTTGAA
TET1	GATCTGTAGGCTCAGGAGGTGTAT	GTATGTAATGCGCCTCTTCCTCT
TET2	CAGTGTATAAGAAAATGGCACCTG	CTCGCGATTATCCTCTCTTGTTAG
TET3	CTAGCAGAAACTCCAGAGAAGACC	GTAACCATGTATGGCTCCATTG
ACTB	CTAAAAACTGGAACGGTGAAGG	AGGCAAATAAGTTTCGGAACAA
EEF1A1l1	AGATGCCGCCATTGTTGAGA	CTTTGTGACCTTGCCAGCAC
Human		
DNMT1	CCTAGCCCCAGGATTACAAGG	ACTCATCCGATTTGGCTCTTTC
DNMT3A	CCGATGCTGGGGACAAGAAT	CCCGTCATCCACCAAGACAC
DNMT3B	AGGGAAGACTCGATCCTCGTC	GTGTGTAGCTTAGCAGACTGG
TET1	CAGAACCTAAACCACCCGTG	TGCTTCGTAGCGCCATTGTAA
TET2	GGCTACAAAGCTCCAGAATGG	AAGAGTGCCACTTGGTGTCTC
MDH-1	TCAGTTGCTTGACTCGTTTGG	GCAATTTCACCTTGGCATGG
EEF-2	GCTCTACCAGACTTTCC	GTCAAAGTACCTGTCACC

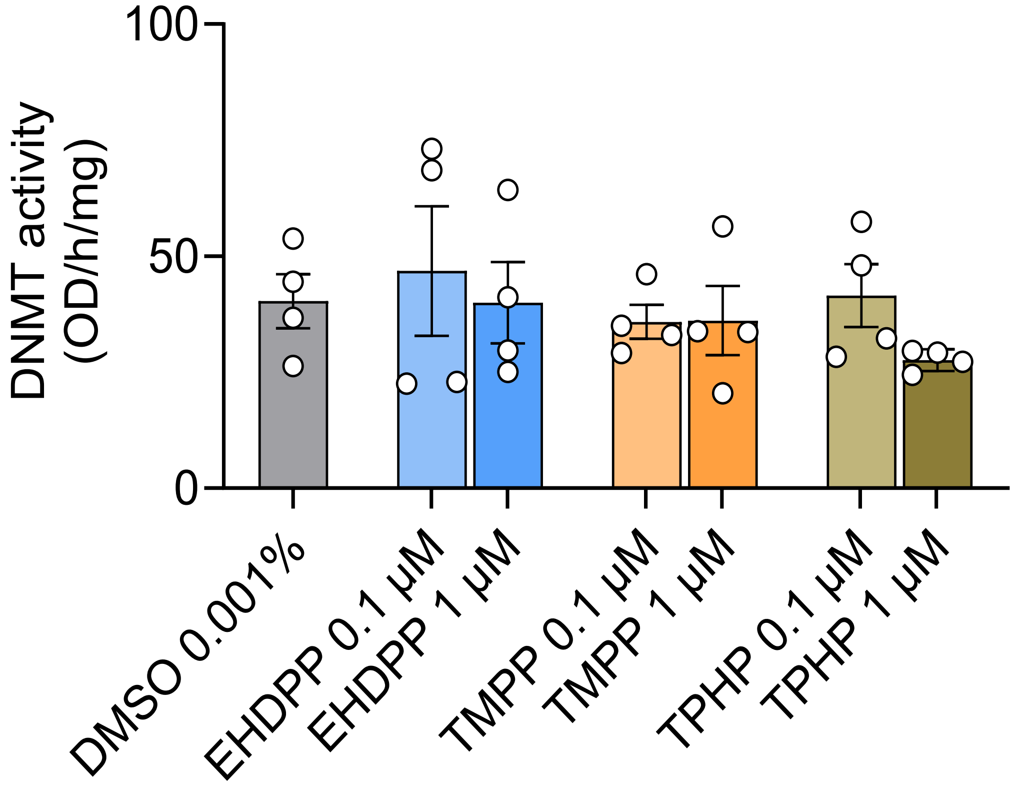

Figure S1. Effect on DNA methyltransferase activity of zebrafish larvae exposed to EHDPP, TMPP, and TPHP. Data represent mean ± SEM of three independent experiments (n=2).

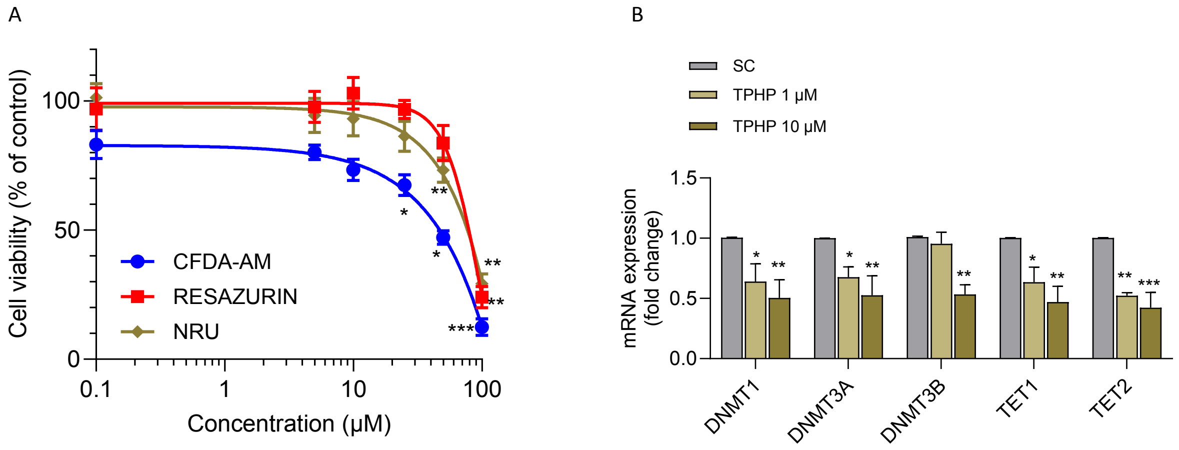

Figure S2. (A) Viability of human liver cells HepG2 treated for 24 h with different concentrations of TPHP as evaluated by CFDA-AM, Resazurin, and Neutral Red Uptake assay, All data normalized to solvent controls (0.1 % v/v DMSO) and presented as mean ± SEM of two independent experiments (n=2) (B) Effect on transcription of genes related to DNA methylation DNMT1, DNMT3A, DNMT3B, TET 1, and TET 2 after exposure of human liver cell culture (HepG2) to TPHP for 24h. Data represent mean ± SEM of three independent experiments (n=3). The asterisks indicate a significant difference from the control at $p < 0.05$ (*), $p < 0.01$ (**), $p < 0.001$ (***).
